# Deletion/loss of bone morphogenetic protein 7 changes tooth morphology and function in *Mus musculus:* implications for dental evolution in mammals

**DOI:** 10.1098/rsos.170761

**Published:** 2018-01-03

**Authors:** Chelsey Zurowski, Heather Jamniczky, Daniel Graf, Jessica Theodor

**Affiliations:** 1 Biological Sciences, University of Calgary, 2500 University Drive NW, Calgary, Alberta, Canada T2N 1N4; 2 Cell Biology and Anatomy, University of Calgary, 3330 Hospital Drive NW, Calgary, Alberta, Canada T2N 4Z6; 3 Dentistry and Medical Genetics, University of Alberta, 116 Street and 85 Avenue, Edmonton, Alberta, Canada T6G 2R3

**Keywords:** dental morphology, dental function, BMP7, inhibitory cascade, geometric morphometrics

## Abstract

Quantifying regulatory gene effects on dental morphology and function has implications for the underlying mechanisms that generated dental diversity in mammals. We tested the hypothesis that regulatory gene expression changes lead to differences in molars using a neural crest knockout of bone morphogenetic protein 7 (BMP7) in *Mus musculus*. Three-dimensional geometric morphometric methods were used to quantify the shape of the molar toothrow. BMP7 mutants have extra cusps on the first upper and lower molars, and alterations in cusp orientation and morphology. Furthermore, significant shape differences between control and mutant were found for upper and lower toothrows. Mutant mice also exhibited differences in attrition facets, indicating functional changes that could lead to advantages in chewing new food resources and eventually niche diversification. The size ratio of the molars in the toothrow remained unchanged, implying that BMP7-induced changes in molar size ratio are a result of knocking out epithelial, rather than neural crest, expression of BMP7. Our results indicate that changes in BMP7 expression are sufficient to alter the morphology and function of the toothrow, suggesting that BMP7 or genes affecting its function could have played a role in structuring the dental diversity of extinct and extant mammals.

## 1. Introduction

Dental characters, such as the shape and size of the molars and their cusps, reveal aspects of phylogeny, taxonomy and ecology that inform most of what is known about mammalian evolution [1,2]. The clade is particularly notable for the widespread presence of convergent dental morphologies in both fossil and recent forms [3], making mammalian teeth an intriguing model for the study of the developmental-genetic architecture of adaptive change [4]. Previous work has elucidated the occurrence, timing and influence of regulatory genes throughout tooth development, the effects of such genes on the final morphology of molars and the relationship of extant molar forms to the forms found in extinct taxa [5–10]. While the relationship between form and function has important evolutionary implications, there remains a substantial gap in our understanding of the functional outcomes of morphological variation in mammal molars.

Bone morphogenetic proteins (BMPs) are a family of regulatory genes that belong to the transforming growth factor β family of extracellular signalling molecules, and that are active throughout tooth development [11] upstream of the Smad, MAPK and PI3 K/Akt pathways [12]. Bone morphogenetic protein 7 (BMP7) is expressed throughout mouse tooth development in both the epithelium and the underlying neural crest-derived mesenchyme [12]. BMP7 null mice exhibit severe craniofacial and dental malformations and die shortly after birth, while BMP7 heterodeficient mice show molar size ratio differences and morphological differences in their dentition [12–15]. Here, we test the hypothesis that BMP7 conditional knockout mice demonstrate both phenotypic and correlated functional changes stemming from the reduction of neural crest-derived BMP7.

## 2. Material and methods

### 2.1. Specimens

Previously collected specimens of control and 1245fl/fl:gTomtg/+:wnt1Cre+ (BMP7Δ) mice, background strain 129SvJ/C57BL/6 [12,16] backcrossed to C57Bl/6 J for more than eight generations, were used in this study. Bmp7^tm1.1Dgra^ mice backcrossed to more than 10 generations of C57Bl6/J were crossed to wnt1-Cre [16] to induce neural crest-specific deletion of Bmp7 (Bmp7^ncko^).

All specimens were fed and housed under identical conditions. Specimens were sacrificed and their heads were preserved. A total of 24 control and 26 mutant specimens were either frozen fresh at −80°C, or preserved using 1% paraformaldehyde and then washed using phosphate-buffered saline (PBS) and stored at 4°C in PBS. Specimens were distributed among five age sets: 21, 53, 60, 75 and 121 days, and spontaneous death specimens, and will be referred to as 1, 2, 3, 4, 5, and E, respectively.

### 2.2. Scanning

Specimens were scanned using a Skyscan 1173 micro-computed tomography (µCT) instrument (Kontich, Belgium) at the University of Calgary and then reconstructed (NRecon, v. 1.6.6.0; 19.46 µm average pixel size, image size: 2240 × 2240 pixel). Three-dimensional surface models of the skulls and the upper and lower toothrows were produced using Amira and exported as polygon model files (.ply) (v. 5.6.0, FEI Visualization Sciences, Hillsborough, OR, USA).

### 2.3. Landmarking and statistical analysis

**Figure 1.**
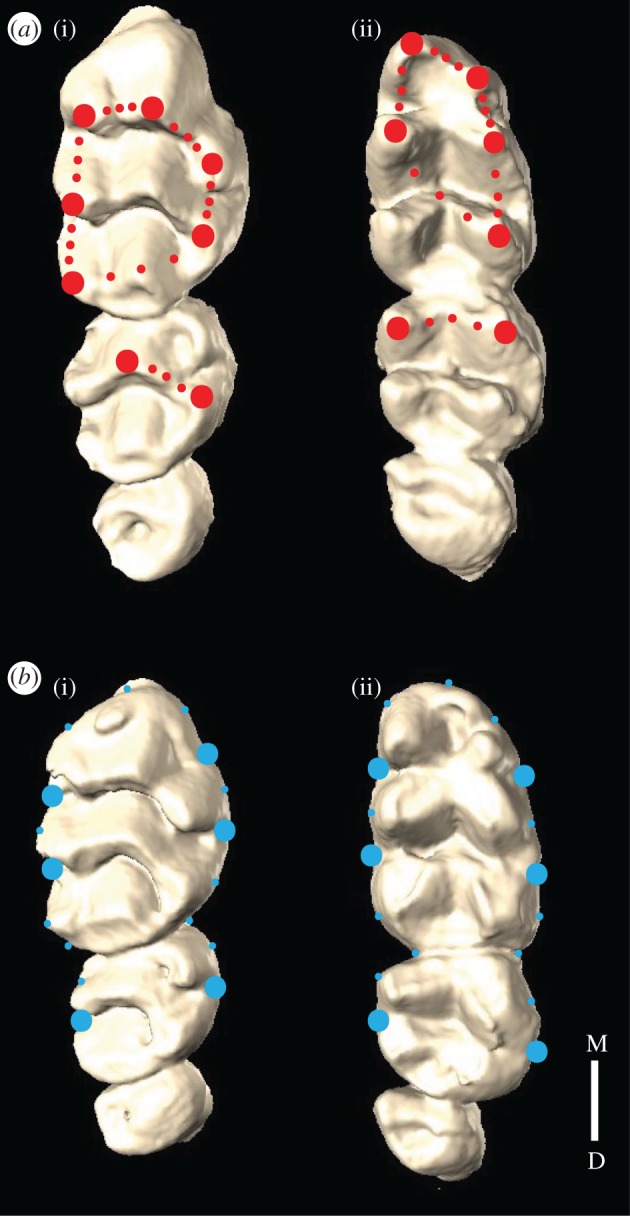
The location of the fixed (large circles) and sliding semilandmarks (small circles) of the cusp (red) *(a)* and outline (blue) *(b)* landmark sets shown on control upper (i) and lower (ii) right toothrows. M, mesial; D, distal.

Landmark-based geometric morphometric methods were used to quantify morphological variation within and between control and mutant toothrows. Two landmark datasets were developed to capture both cusp and outline morphologies, and consisted of both fixed and sliding semilandmarks (figure 1; electronic supplementary material, SI1). Each specimen was landmarked three times for each set by the same observer on different days in order to quantify intra-observer error, and these replicates were then averaged and subjected to Procrustes transformation for analysis. Principal components analysis (PCA) was performed to determine the major axis of variation present in each dataset [17]. Differences in shape between mutant and control specimens were quantified using a morphological disparity test and discriminant function analysis (DFA). Morphological disparity estimates group variance and through permutation estimates statistical significance of between-group shape variance [18]. DFA describes shape differences between groups by the linear axis of greatest variation [19]. The first and third semilandmarks between the fixed landmarks were excluded in the cusp landmark sets to accommodate the requirements of fewer variables than specimens in the DFA. Finally, to examine the relationship between size and shape, a regression of shape score on centroid size was performed for each transformed dataset [20]. All landmarking and statistical analyses were executed using the Geomorph package (v. 3.0.1; [21]) in RStudio (v. 00.99.893, R v. 3.3.0 GUI 1.68 Mavericks build, R Core Team 2016, [22]).

### 2.4. Wear facets

Two mutant and two control specimens from age sets 1, 3 and 5 were skinned and skeletonized following scanning. Photographs of the right lower toothrows were taken using a Dino-Lite Edge imaging system (AM4815ZTL Dino-Lite Premier; AnMo Electronics Corporation, Taiwan) and the wear facets were subsequently manually outlined in Adobe Illustrator CS6 (v. 16.0.3). Facet morphology was then qualitatively compared.

### 2.5. Description

A detailed description of the mutant specimens, along with additional figures, can be found in the electronic supplementary material, SI1.

## 3. Results

**Figure 2.**
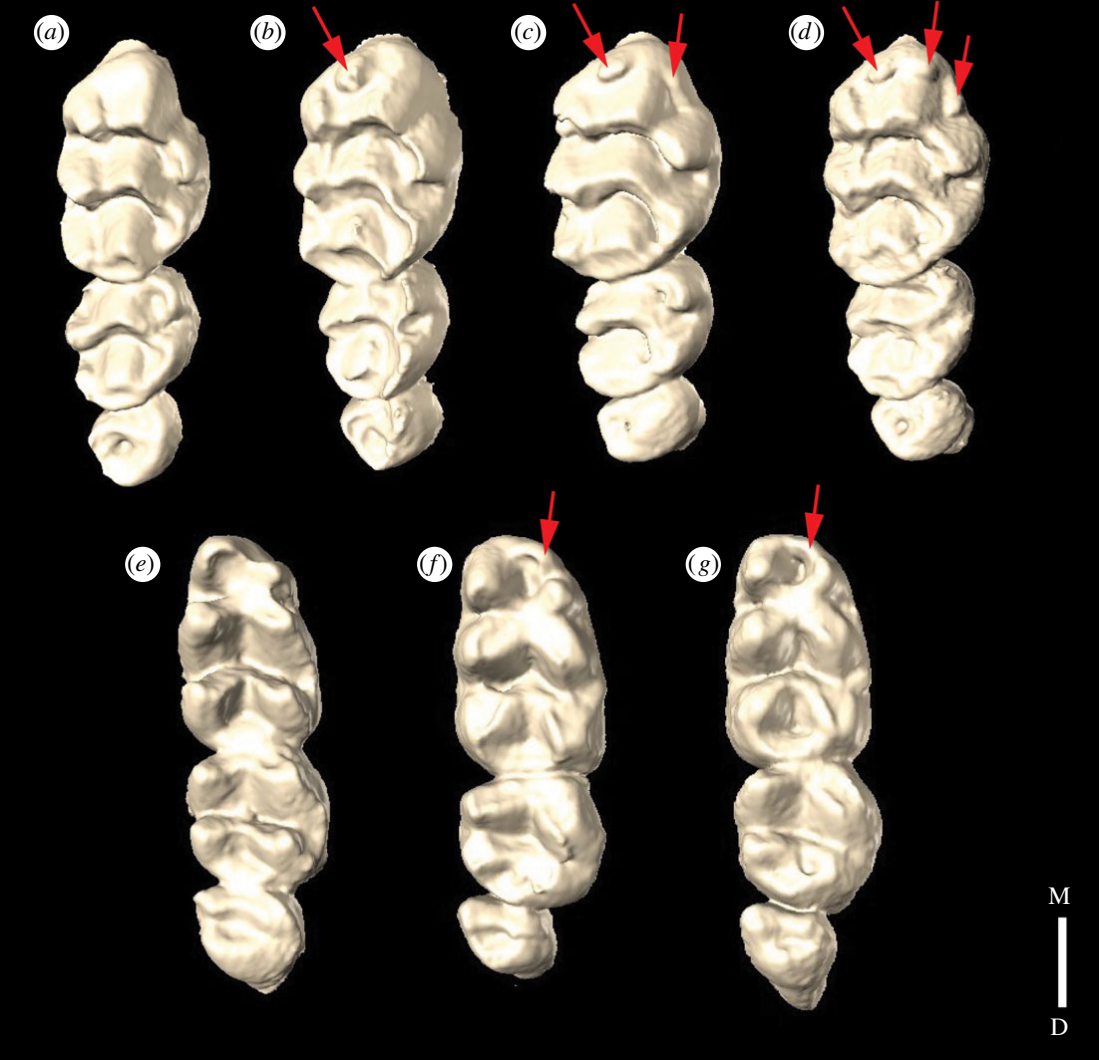
Comparison of control upper (*a*) and lower (*e*) toothrows with BMP7 conditional knockout upper toothrow morphotypes (*b*–*d*) and lower toothrow morphotypes (*f*,*g*). Red arrows point to accessory cusp(s) on the mutant toothrows. M, mesial; D, distal.

Mutant molars were short and broad both mesiodistally and buccolingually and were observed to have extra cusp(s) on the first upper and lower molars (figure 2; electronic supplementary material, SI1). Multiple morphotypes of the ‘extra cusp’ morphology were present in both the upper and lower first molars (figure 2; electronic supplementary material, SI1).

**Figure 3.**
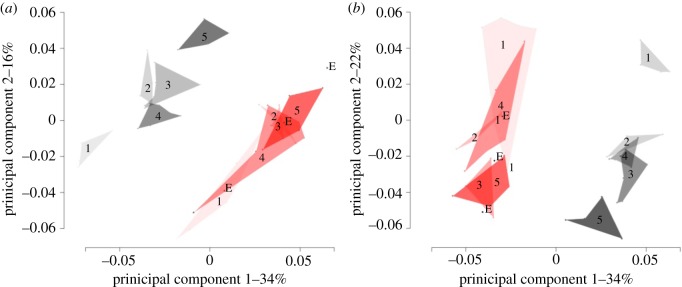
PCA plots for the (*a*) upper and (*b*) lower cusp landmark sets. Controls are pictured in grey and mutants in red with labels for age set and lines delineating the morphospace each age set occupies.

Three-dimensional geometric morphometric analyses confirmed shape differences between mutants and controls in each of the landmark sets and exposed differences in attrition facet development (figure 3). Specimens were clustered by genotype and age set in both upper and lower cusp landmark PCA plots, with the age sets clustering consecutively from youngest to oldest (figure 3). In PCA plots for the upper and lower outline landmark sets, specimens were only clustered by genotype (electronic supplementary material, SI2). Morphological disparity tests revealed significant differences between mutant and control specimens for only the lower cusp landmark set (electronic supplementary material, SI3). For all landmark sets, DFA consistently categorized specimens by genotype, but not by age (electronic supplementary material, S13). Regression analyses returned significant results for only the lower cusp and outline landmark sets (electronic supplementary material, SI3).

**Figure 4.**
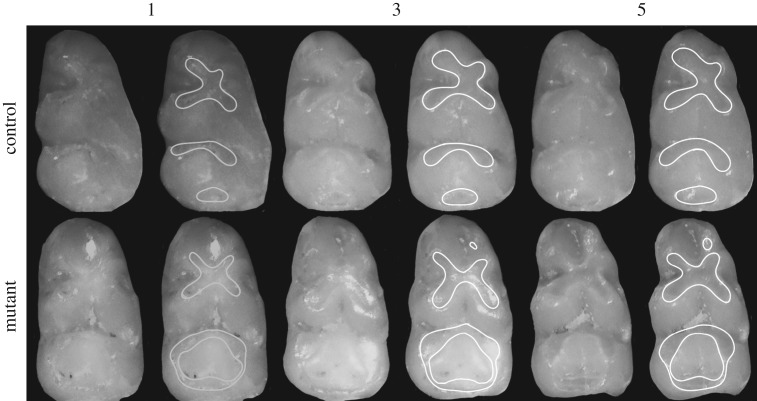
Comparison of wear facet morphology in mutant and control skeletonized specimens from age sets 1, 3 and 5. Each specimen is shown twice, both with and without outlines of the wear facets. Control specimens are pictured on the top row and BMP7 mutants on the bottom row. Age sets are labelled along the top.

Attrition facets on the m1 in mutant mice were visually different in morphology and exhibited accelerated wear progression patterns in all age sets examined (figure 4). In addition to large and broad wear surfaces, there was fusion between the facets on the second chevron and talonid, resulting in a large distal-facing wear surface that merged over the entoconid and metaconid (figure 4).

## 4. Discussion

Neural crest-derived mesenchymal cells contributing to molars express BMP7 in the placode and bell stages of tooth development [12]. This expression occurs in the underlying mesenchyme and the dental papilla, which are precursors to odontoblasts [12]. Reducing the BMP7 expression in these cells resulted in molar morphology that differed from controls using both qualitative and quantitative methods (figure 2; electronic supplementary material, SI1).

### 4.1 Neural crest-derived bone morphogenetic protein 7 and dental morphogenesis in mice

Morphological differences in mutant molars suggest that a lack of mesenchymal BMP7 leads to differences in the distribution and timing of the secondary enamel knot development and/or modifications in differential growth at the enamel dentine junction (EDJ) where the main shape of the tooth is formed [6]. BMPs have been shown to be important in cellular motility and for the ability of cells to form apical and basal surfaces [23,24]. Lack of neural crest-derived BMP7 in mutants could disrupt cellular migration and organization, leading to morphological differences in the EDJ and the final molar shape, and the potential for novel molar form and function. Histological examination during molar development is necessary to determine the effects at the cellular level.

### 4.2 Functional consequences of changes in molar morphology

Cusp morphology is important in understanding the evolution of function in murine molars, and tooth wear is a complex process that greatly influences the shape of the cusps [25,26]. Morphological changes in the first molars are especially important within Murinae, as the first molars are the antagonistic set responsible for the majority of mastication [25]. Additionally, differences in attrition facet shape, size and distribution imply alterations in the way molars occlude, along with changes in molar function [27].

Similar morphologies to those observed here can be observed within many other members of Murinae, in mice with alterations in different regulatory genes, and as a spontaneous morphology in control specimens from other studies [9,28–35]. The occurrence of similar molar form within Murinae suggests the hypothesis that BMP7 may be a source of evolutionary important variation in dentition. For instance, accessory cusps and bigger molars, such as those observed in mutant specimens, can contribute to a large overall wear surface that may allow for processing of different food types [9,36].

Together, mutant molar and facet morphology correlate with the low-cusped molars and broad wear surfaces that are typical of mammalian dietary adaptations to frugivory [37]. This implies that the resulting morphological and functional differences owing to expression changes in BMP7 may lead to advantages in chewing alternate food resources and potentially niche diversification. Further testing with the food of varying hardness is required to confirm the potential to occupy a new niche.

### 4.3 Genomic organization and evolutionary implications

BMPs are highly conserved proteins with activity in many different tissues, which limit the possibility for changes to the protein itself as an evolutionary mechanism. However, it has been shown that the genomic organization of several members of the BMP family is modular. The genes are located in gene deserts that contain numerous long-range *cis*-regulatory enhancer sequences to control cell- and tissue-specific gene expression [38]. There is evidence that BMP7 is regulated in a similar manner [15]. The principle of enhancer modules makes changes to gene expression at a particular site relatively easy without affecting expression at other sites. Differences in BMP7 expression specifically in developing molars could thus have been achieved by such a mechanism.

### 4.4 Alignment with the inhibitory cascade model

The inhibitory cascade model (ICM) is a developmental model that states that mesial molars suppress the size of more distal molars, resulting in a gradient M1 > M2 > M3 [8]. No differences in the molar surface area ratios were observed between BMP7 conditional knockouts, where BMP7 expression was reduced in the neural crest-derived mesenchyme but not in epithelial tissues, and the control mice. Occlusal surface area ratio differences in the molars of BMP7 heterodeficient mice, where both epithelial tissue and neural crest cells in the molars had reduced expression of BMP7 [14], suggest that if the ICM is correct, BMP7 contributes to the size gradient by its expression in epithelial tissues and not in the neural crest.

Although no differences were observed in the molar size ratios, the mesial portion of the molars, especially in the M1, possessed higher amounts of morphological variability. Molar development proceeds sequentially with the M1 developing first, after which the M2 develops from the distally extending dental lamina [8]. In theory, this would allow morphological variation and novel morphologies on the mesial portion of the tooth, as it would be less likely to influence the development of subsequent molars.

## 5. Conclusion

Knockout of neural crest-derived BMP7 causes both morphological and functional changes in mouse teeth, showing that suppression of regulatory genes is a feasible means to generate variation in mammalian dentition. The changes observed here correlate with morphologies observed within Muridae and may correspond to dietary changes that allow for niche diversification.

Further research studies into cell dynamics in the tooth bud, along with the functional consequences of the final morphology in BMP7 conditional knockout mice, are needed to elucidate the relationship between regulatory gene expression patterns and mammalian dental evolution.

## Supplementary Material

Morphological comparisons of teeth and details of morphometric analyses

## Data accessibility

Our data are deposited at Dryad: http://dx.doi.org/10.5061/dryad.70585 [39].
