# Supplementary material for: Deletion/loss of bone morphogenetic protein 7 changes tooth morphology and function in Mus musculus: implications for dental evolution in mammals
Source: R Soc Open Sci. 2018 Jan 3;5(1):170761. doi: 10.1098/rsos.170761 (PMC5792877; doi:10.1098/rsos.170761)
Supplement: Morphological comparisons of teeth and details of morphometric analyses [file rsos170761supp1.pdf]

SI1 – DESCRIPTION

General Craniofacial Description

Mutant skulls were brachycephalic when compared with control specimens. The parietal and occipital regions of the skull were expanded and bulbous, making the dorsal and posterior portions of the skull rounded (SI2). There was a spectrum in the amount of brachycephaly present in mutant specimens, with some mutants having a higher degree of brachycephaly than others. The rostrum was short and sloped ventrally, whereas the slope in the control skulls the rostrum was not as strong (SI2). The frontal bones in the mutant skulls were anteroposteriorly short and did not reach posteromedially as they did in the control skulls, and instead were parallel to the coronal plane (SI2). The mutant nasal bones were transversely broad, upturned anteriorly, and ended abruptly without extending to the length of the anteriormost point of the upper incisors. In dorsal view, the zygomatic arch curved medially, and in lateral view it dipped ventrally. The intraorbital distance was expanded, as was the width of the rostrum. In lateral view the maxilla did not extend anterodorsally, but was parallel with the visible portion of the upper incisors. The squamosal was small, and the ridge that usually runs between the squamosal and the parietals was not present. Mutant mandibles were short anteroposteriorly, wide buccolingually, the lower toothrow sloped slightly anteroventrally and the condyle and coronoid process flared laterally.

General Dentition Description

The section below describes specific morphology differences observed in each tooth when comparing the mutant and control tooth morphology. All mutant teeth had cusps height that was shorter and broader both mesiodistally and buccolingually. Variable wear patterns were observed in the mutant molars, but will be described more specifically by tooth below.

Mutant Lower Molars

Mutant First Molar (m1)

The first molar showed a higher degree of within group variability in mutant mice compared to the second and third molars, especially on the mesial surface of the tooth. Mesial to the labial anteroconid there was a small accessory cusp in all but one of the mutant specimens. This, in addition to a more mesially located labial anteroconid, made the mesial outline surface of the tooth appear squared. The extra cusp on the mesial aspect was either present directly mesial to the labial anteroconid, or the accessory cusp was positioned mesiolingual to the labial anteroconid and mesiobuccal to the lingual anteroconid, these two morphotypes were equally common (Figure 2). When the extra cusp was mesial to the valley between the anteroconids it was roughly incorporated into the occlusal surface of the anterior chevron (Figure 2), and when the cusp was mesial to the labial anteroconid there was a valley between these two cusps and the lingual anteroconid (Figure 2). Rather than pointing dorsally, the cusps on the mesial surface of the tooth were slightly posteriorly recurved. The cusps on the toothrow were individualized and presented less ridging between cusps. This was present in all three chevrons.

The cusps on the distal portion of the tooth had variable wear patterns that were present more commonly in the older age sets, where the wear facets had more time to develop. The entoconid commonly possessed variable morphologies from a mesial or distal view, including a stepped morphology and an M-shaped morphology with the center of the cusp, where the maximum point of the cusp is usually present, dipping ventrally. The hypoconid also had this stepped morphology, and was commonly almost worn off.

Mutant Second Molar (m2)

The shape of the mutant second lower molar was rhomboid, as compared with the square shaped second molar in control specimens. The mesial margin of the m2 did not run parallel to the distal margin of the m1 and instead, the metaconid-protoconid chevron was triangular, with the point of the triangle created by the medial portion of the protoconid, directed mesiobuccally towards the hypoconid on the m1. In general the cusp sizes were variable but the mesial cusps (i.e. protoconid and metaconid) tended to be larger than the distal cusps (i.e. hypoconid and entoconid). In many cases, especially in the older age sets, the hypoconid was nearly worn flat (Figure 2).

Mutant Third Molar (m3)

The third molars were located lingually on the toothrow in relation to the mesial teeth. Mutant teeth were shorter mesiodistally, and the mesial margin of the tooth was buccolingually wider. Unlike the controls, the two small cusps on the mesial side of the tooth were not even in size. The metaconid was large and contributed to half of the overall tooth size, whereas the protoconid was very small. The distal portion of the tooth came to a point, making the outline shape of the mutant m3 triangular, rather than the rhomboid shape observed in control specimens (Figure 2).

Mutant Upper Molars

Mutant First molar (M1)

Mutant first upper molars were broader buccolingually than those of the control mice and their outline shape was squared. Similar to the lower first molar, the upper first molar was most variable in the cusp morphology mesial on the M1. Out of the 25 mutant specimens, fourteen of them had three

extra upper mesial cusps, seven had two, and four had one (Figure 2). Specimens with one extra cusp usually had it placed mesial to the valley between t2 and t3, but one specimen had it present in the notch between t1 and t2 (Figure 2). In specimens with two extra cusps, the cusps were located either with both mesial to t2 and t3, or one mesial to t2 and t3 and one in the notch between t1 and t2 (Figure 2). In the specimens that had three extra cusps, one cusp is located mesial to t2, one mesial to the valley between t2 and t3, and the third cusp is in the concave notch of the outline mesial to the space between t1 and t2 (Figure 2). The addition of these mesial cusps contributed to differences in shape of the mesial outline of the tooth, and they were present across all age sets. The mutant first chevron, t1, t2 and t3, was positioned further distally from the mesial outline edge of the tooth, compared with the location of the chevron in control molars. As with the lower molar morphology, the ridges between the cusps of the tooth were less defined and the cusps were more individualized, meaning that they are not as connected to other cusps via ridges. The anterior lingual cusps of the tooth, t1 and t4, were shifted away from one another, with t1 positioned anteriorly and t4 positioned distally. The posterior aspect of the tooth and the t8 were wider buccolingually.

Mutant Second Molar (M2)

The second molar in the upper row, like the second molar in the lower row, was positioned lingually when compared with the first molar. t4 was shifted distally, which increased the amount of separation between t1 and t4. Rather than the t4, t5 and t6 chevron forming a triangular shape with the tip facing mesially, as seen in controls, t5 was located distally and t6 mesially, making the ridge parallel with the mesial surface of the tooth. The buccal cusps, t6 and t9, were positioned mesially. In some specimens, the maximum of the t1 cusp recurved distally. Additionally, t8 was flattened, broadened, and almost completely worn in some of the specimens from older age sets.

Mutant Third Molar (M3)

The M3 was positioned lingually in the toothrow in comparison with M1 and M2. The tooth was wider buccolingually than it was mesiodistally, compared to the equilateral triangle-shape of the control M3. The t8 extended lingually and mesially, towards t4 on the M2.

SI2 – ADDITIONAL FIGURES

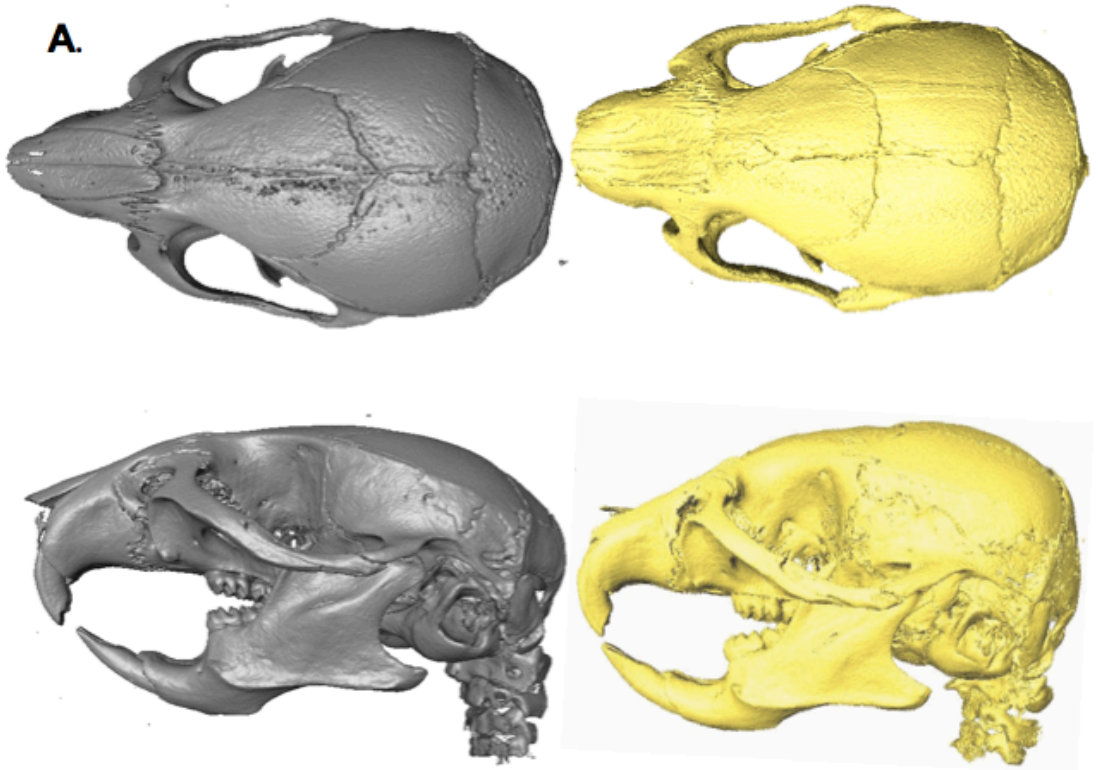

SI2 Figure 1: Comparison of 3D models of control (grey) and mutant (yellow) skulls in dorsal and left lateral views.

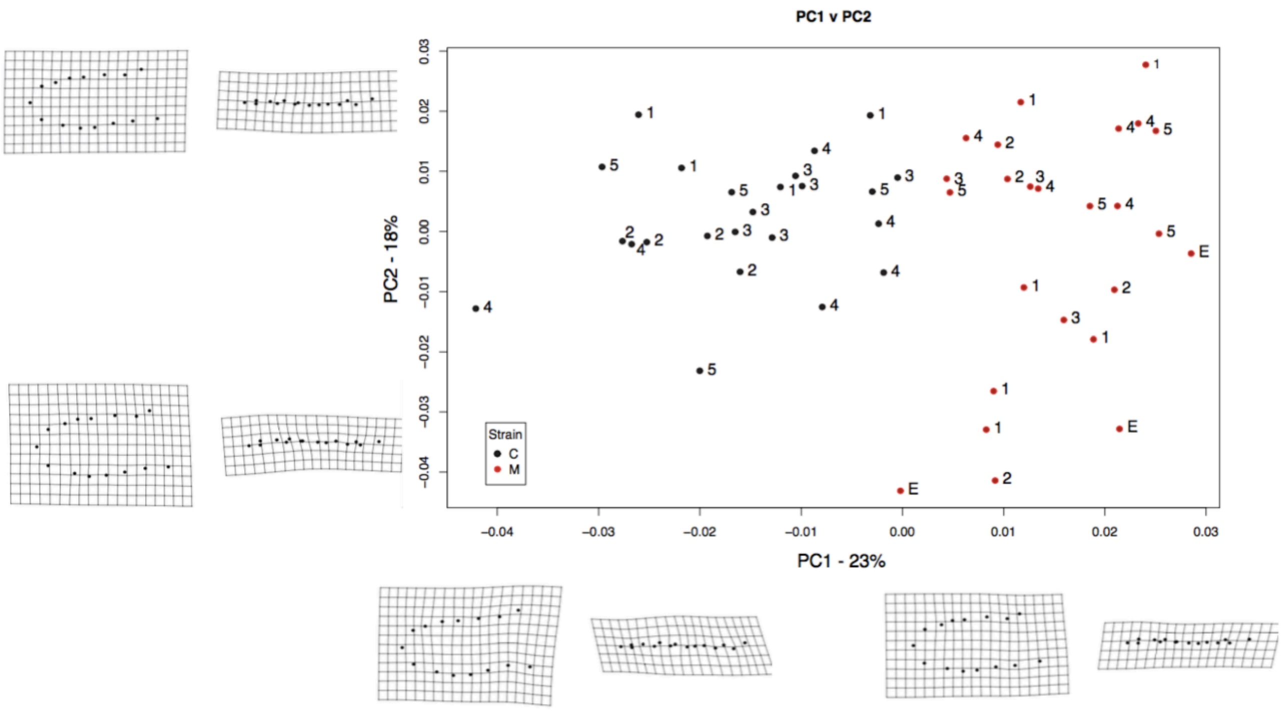

SI2 Figure 2: PCA plot for the averaged Lower Outline landmark set. Black points show control specimens and red points show mutant specimens. The number beside each point corresponds to the age set that the specimen belongs to. Warpgrids show the average shape of the landmark configuration at PC1 and PC2 maximum and minimum.

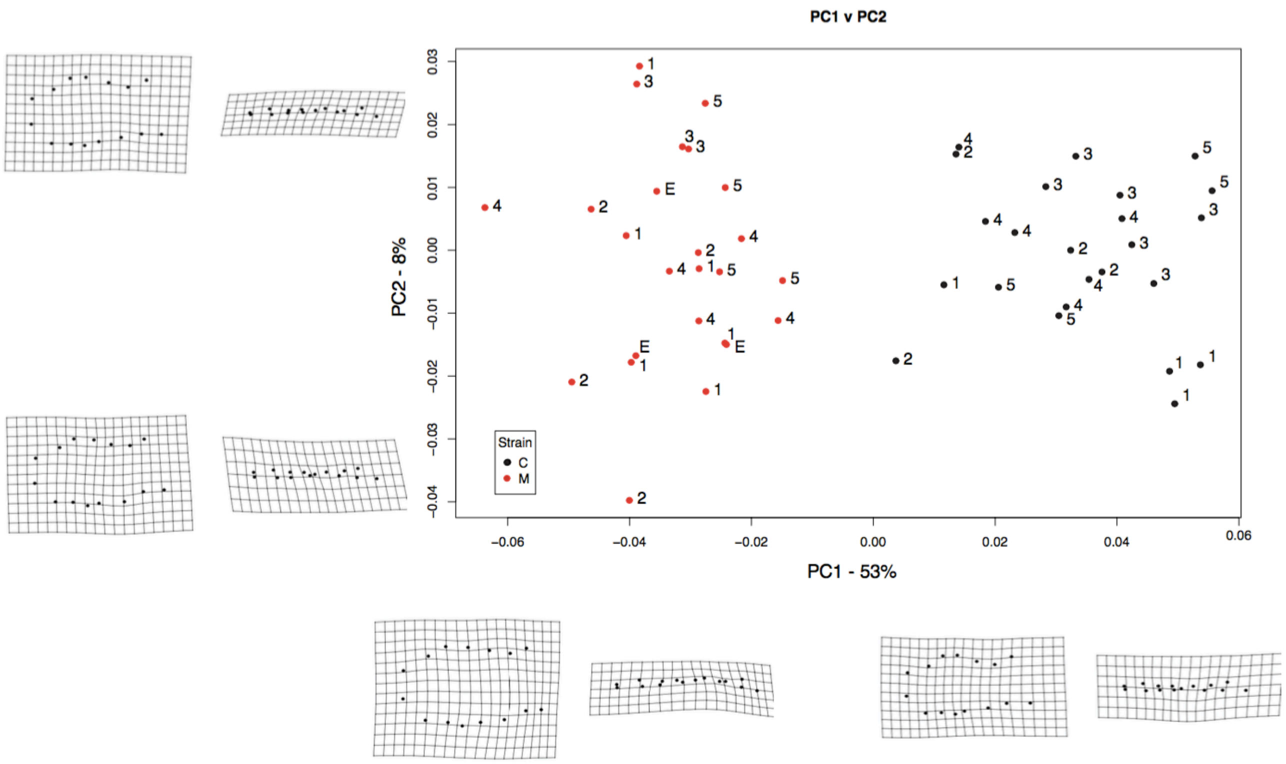

SI2 Figure 3: PCA plot for the averaged Upper Outline landmark set. Black points show control specimens and red points show mutant specimens. The number beside each point corresponds to the age set that the specimen belongs to. Wargrids show the average shape of the landmark configuration at PC1 and PC2 maximum and minimum.

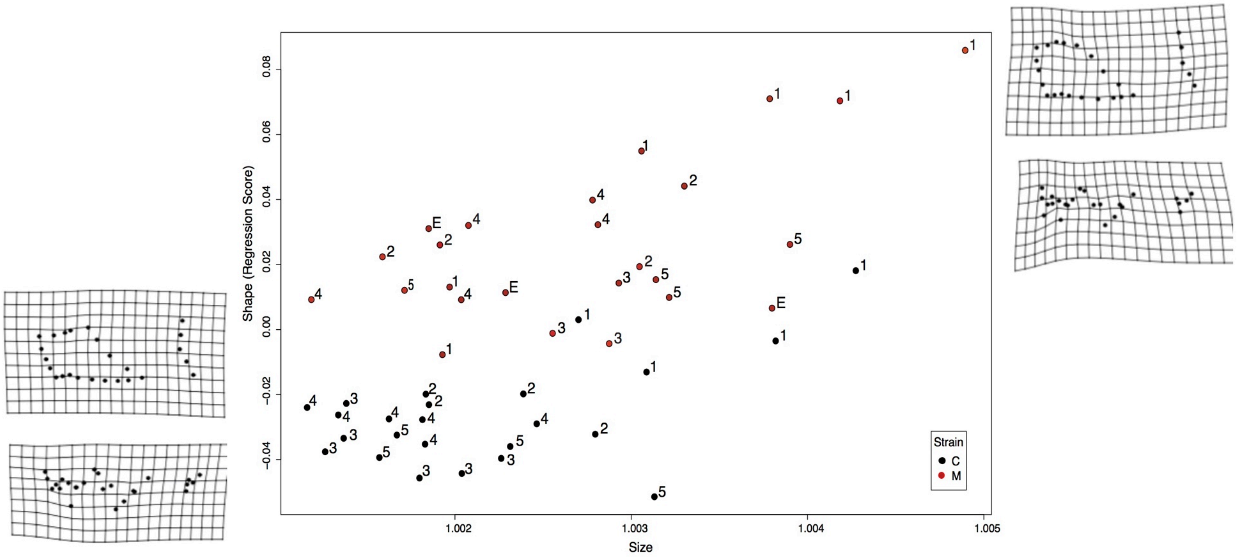

SI2 Figure 4: Regression of shape on size of the Lower Cusp landmark set. Black points are control specimens and red points are mutant specimens. Numbers beside the points correspond to the age set that the specimen belongs to. Warpgrids show the shape of the landmark configurations at maximum and minimum centroid size.

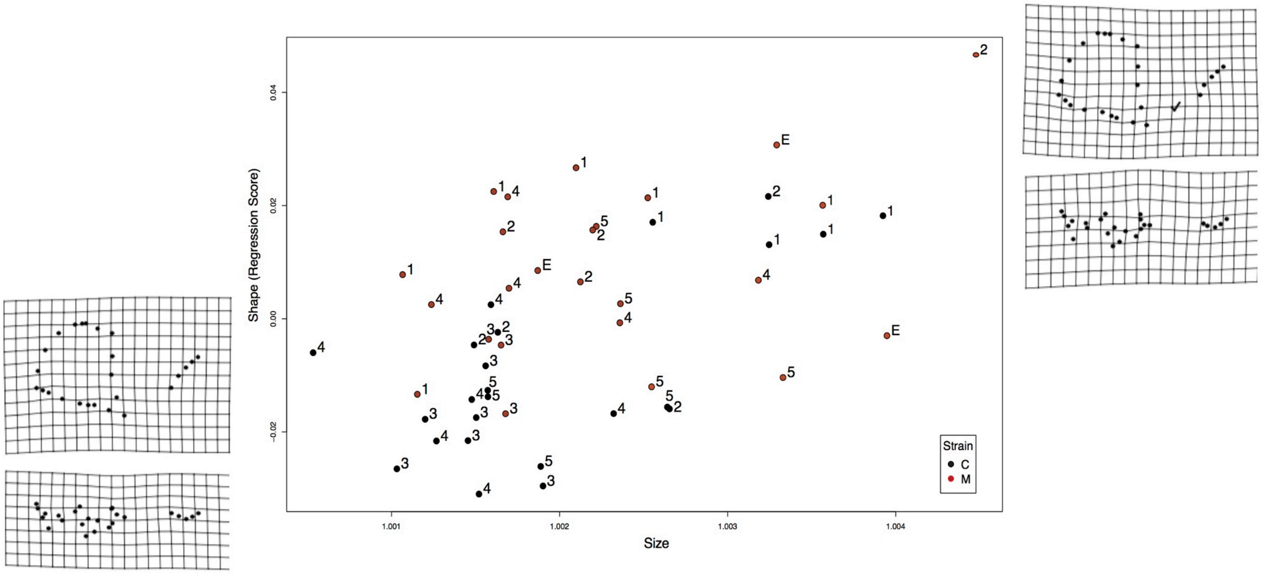

SI2 Figure 5: Regression of size on shape of the upper cusp landmark set. Black points correspond to control specimens and red points correspond to mutant specimens. Numbers beside specimen points indicate the age set that the specimen belongs to. Warpgrids show the shape differences of the landmark configuration at maximum and minimum centroid size.

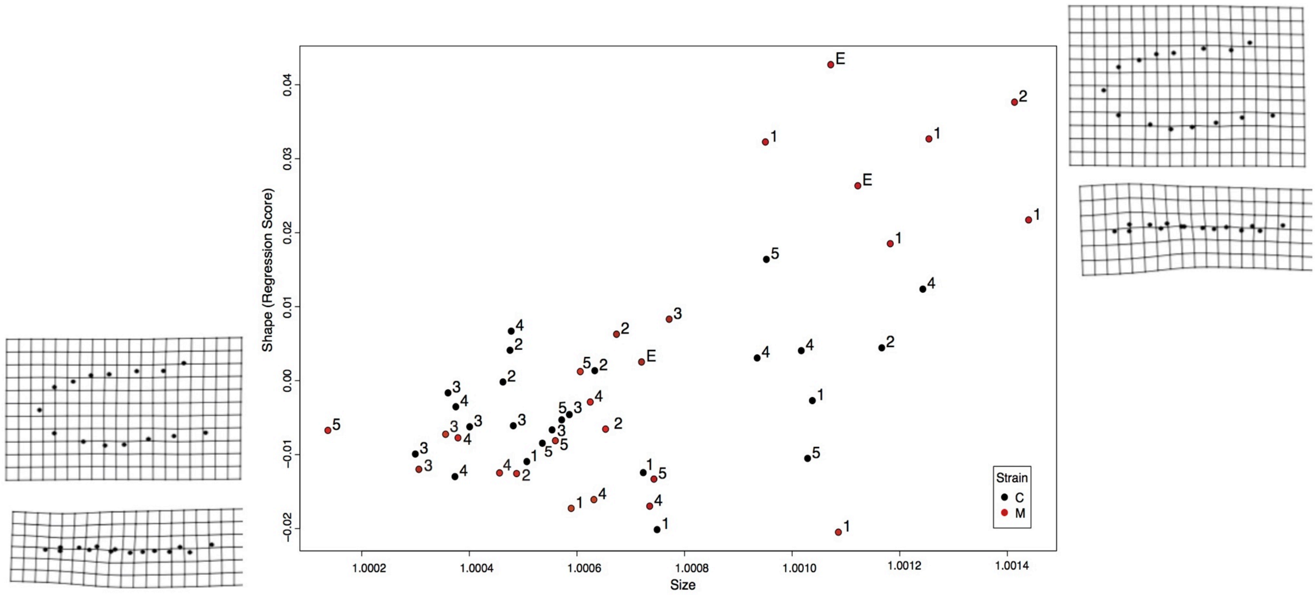

SI2 Figure 6: Regression of shape on size of the Lower Outline landmark set. Black points correspond to control specimens and red points correspond to mutant specimens. Numbers beside each point correspond to their age set. Warpgrids show the shape differences at minimum and maximum centroid size.

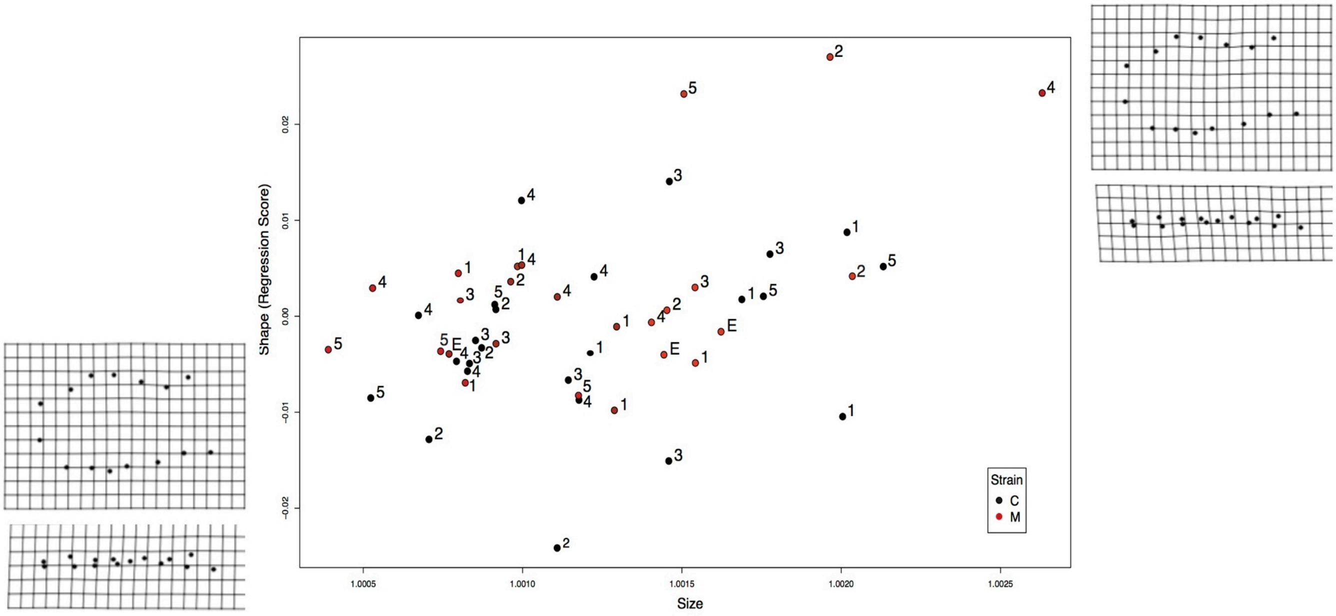

SI2 Figure 7: Regression of shape on size of the upper outline landmark set. Black points correspond to control specimens and red points correspond to mutant specimens. Numbers beside each point indicate the age set they belong to. Warpgrids show the shape differences at maximum and minimum centroid size.

SI3- RESULTS TABLES

Table 1. Mus musculus specimens employed in this study, classified according to age class, genotype, and sex.

Age Class	Total	Number of Female Controls	Number of Male Controls	Number of Female Mutants	Number of Male Mutants
1	10	1	3	2	4
2	8	0	4	2	2
3	9	3	3	2	1
4	11	4	2	1	4
5	8	0	4	0	4
E	4	0	0	2	2
Total	50	8	16	9	17

Table 2: Summary of Procrustes ANOVA results on replicate landmark data for each landmark set.

Landmark Set	F	R^2	p
Cusp Lower	0.623	0.00864	0.884
Cusp Upper	0.826	0.0113	0.674
Outline Lower	1.47	0.0201	0.0769

**Outline Upper**	0.988	0.0135	0.404

Table 3: Percentage of variation explained by Principal Components 1 and 2 (PC1 and PC2) for each landmark set.

**Landmark Set**	**PC1 (%)**	**PC2 (%)**
**Cusp Lower**	33.9	22.2
**Cusp Upper**	33.9	16.2
**Outline Lower**	22.5	18.3
**Outline Upper**	52.9	8.41

Table 4: Summary of Regression analysis by Procrustes ANOVA of shape variation on centroid size for each landmark set.

**Landmark Set**	**F**	**R^2**	**Pr (>F)**
**Cusp Lower**	4.0278	0.078934	0.001889
**Cusp Upper**	0.9674	0.0041497	0.4348
**Outline Lower**	3.2139	0.064004	0.002997
**Outline Upper**	0.301	0.0063637	0.954

Table 5: Percentage of correctly identified specimens by Discriminant Function Analysis when using strain as the grouping variable for each landmark set.

Landmark Set	Control	Mutant	Total correctly classified (%)
**Cusp Lower**	100	100	100
**Cusp Upper**	87.5	72.0	80
**Outline Lower**	100	96.0	98
**Outline Upper**	95.8	84.0	90

Table 6: Specimens correctly identified by Discriminant Function Analysis when using age set as the grouping variable shown as a percentage.

Age Set	Percentage Correctly Identified (%)						
	1	2	3	4	5	E	Total Correctly Classified (%)
**Cusp Lower**	20.0	12.5	11.1	9.09	12.5	66.7	16.3
**Cusp Upper**	40.0	25.0	0.00	0.00	62.5	33.3	24.5
**Outline Lower**	30.0	12.5	11.1	9.09	37.5	0.00	18.4
**Outline Upper**	20.0	37.5	22.2	0.00	25.0	33.3	20.4

Table 7: Procrustes variances from a Morphological Disparity test using strain as the grouping factor. Test was performed on each landmark set.

Landmark Set	Control	Mutant	Differences between variances
**Cusp Lower**	0.004286	0.005430	$p<0.05$
**Cusp Upper**	0.003883	0.004502	$p>0.05$
**Outline Lower**	0.001307	0.001493	$p>0.05$
**Outline Upper**	0.002396	0.002437	$p>0.05$

Table 8: Procrustes variances from a Morphological Disparity test for each age set and landmark set.

Landmark Set	Procrustes Variance for Each Age Set					
	**1**	**2**	**3**	**4**	**5**	**E**
**sCusp Lower**	0.006694	0.004640	0.004041	0.003802	0.005087	0.005222
**Cusp Upper**	0.004978	0.004787	0.002965	0.003390	0.00446	0.005998
**Outline Lower**	0.001870	0.001473	0.0008954	0.001296	0.001271	0.001918
**Outline Upper**	0.002694	0.002482	0.002374	0.002230	0.002267	0.002534
